# Does the Somatosensory Temporal Discrimination Threshold Change over Time in Focal Dystonia?

**DOI:** 10.1155/2017/9848070

**Published:** 2017-09-14

**Authors:** Antonella Conte, Gina Ferrazzano, Daniele Belvisi, Nicoletta Manzo, Antonio Suppa, Giovanni Fabbrini, Alfredo Berardelli

**Affiliations:** ^1^Department of Neurology and Psychiatry, Sapienza University Rome, Rome, Italy; ^2^IRCCS Neuromed, Pozzilli, Italy

## Abstract

**Background:**

The somatosensory temporal discrimination threshold (STDT) is defined as the shortest interval at which an individual recognizes two stimuli as asynchronous. Some evidence suggests that STDT depends on cortical inhibitory interneurons in the basal ganglia and in primary somatosensory cortex. Several studies have reported that the STDT in patients with dystonia is abnormal. No longitudinal studies have yet investigated whether STDT values in different forms of focal dystonia change during the course of the disease.

**Methods:**

We designed a follow-up study on 25 patients with dystonia (15 with blepharospasm and 10 with cervical dystonia) who were tested twice: upon enrolment and 8 years later. STDT values from dystonic patients at the baseline were also compared with those from a group of 30 age-matched healthy subjects.

**Results:**

Our findings show that the abnormally high STDT values observed in patients with focal dystonia remained unchanged at the 8-year follow-up assessment whereas disease severity worsened.

**Conclusions:**

Our observation that STDT abnormalities in dystonia remain unmodified during the course of the disease suggests that the altered activity of inhibitory interneurons—either at cortical or at subcortical level—responsible for the increased STDT does not deteriorate as the disease progresses.

## 1. Introduction

Dystonia is a movement disorder characterized by sustained or intermittent muscle contractions that cause abnormal, often repetitive, movements and postures [1, 2]. Depending on its distribution in the body, dystonia is classified under generalized, segmental, and focal forms, with the last being the most common in adult patients [1, 2]. Although the underlying pathophysiological mechanisms of dystonia are still debated, a large body of evidence suggests that reduced inhibitory activity at various levels of the central nervous system and altered cortical plasticity are involved [3–8].

The somatosensory temporal discrimination threshold (STDT) is the shortest interval at which an individual recognizes a pair of stimuli as separated in time [9, 10], and previous studies have shown that the STDT depends on the integrated activity of an extensive network that includes sensory cortex and basal ganglia [11–14]. Consistent findings have also shown that the STDT in patients with focal and generalized dystonia is abnormal [15–18].

Since the STDT is abnormally increased in both affected [16, 17, 19, 20] and unaffected body regions [21] in patients with dystonia as well as in patients' unaffected relatives [16, 17, 21], the STDT has been proposed as a mediational endophenotypic feature of dystonia [14].

In healthy subjects, inhibitory interneurons in primary somatosensory cortex play a role in STDT by sharpening and focusing sensory information in the temporal domain [13, 22]. Several authors have suggested that an abnormal activity of inhibitory interneurons in S1 is likely to be responsible for the increased STDT values in dystonia [23–25]. No longitudinal studies have yet investigated whether STDT values in focal dystonia change during the course of the disease. A better understanding of this issue may shed light on the pathophysiological mechanisms underlying STDT abnormality. To this end, we designed a follow-up study to investigate whether the STDT values of patients with different forms of focal dystonia change during the course of the disease. For this purpose, we tested a group of patients with focal dystonias (blepharospasm and cervical dystonia) twice: the first time upon enrolment and the second time 8 years later. STDT values from dystonic patients at baseline were compared with those from a group of age-matched healthy subjects. We also investigated possible correlations between changes in STDT values and changes in disease severity.

## 2. Methods

Twenty-five patients with primary focal dystonia (15 patients with blepharospasm and 10 patients with cervical dystonia) (Table 1) were enrolled in the study from the outpatient clinic of movement disorders, Department of Neurology and Psychiatry, Sapienza, University of Rome. Thirty age-matched healthy subjects (age: 59 ± 13 years) were enrolled as controls. All the dystonic patients were studied 4 months after the last botulinum toxin injection. Information regarding the patients' demographic features, medical and family histories, disease course, and treatment were collected during a face-to-face interview (Table 1). Since STDT testing assesses a psychometric function, it yields reliable data only in the absence of cognitive or overt psychiatric conditions. Exclusion criteria for this study were therefore a medical history of psychiatric conditions and those patients with a FAB score lower than 15. To rate disease severity, we used a three-point clinical scale (1 = mild, 3 = severe) to assess the clinical severity for blepharospasm [15, 19] and the Toronto Western Spasmodic Torticollis Rating Scale (TWSTRS) [26] for cervical dystonia. The study was approved by the local institutional review board and performed in accordance with the Declaration of Helsinki.

The STDT was investigated by delivering paired stimuli starting with an interstimulus interval of 0 ms (simultaneous pair), and progressively increasing the interstimulus interval in 10 ms steps, according to the experimental procedures used in the previous studies [15, 19, 27–29]. Paired tactile stimuli consisted of square wave electrical pulses delivered with a constant current stimulator (Digitimer DS7AH) through surface skin electrodes with the anode located 0.5 cm distally to the cathode. Since the body part affected by dystonia in blepharospasm and cervical dystonia is different, we tested STDT values on the volar surface of the right index finger in order to obtain a between-group comparison of STDT values on the same body part. The stimulation intensity was defined for each subject by delivering a series of stimuli at an intensity that increased in 0.5 mA steps starting from 2 mA; the intensity used for the STDT was the minimum intensity perceived by the subject in 10 of 10 consecutive stimuli. The first of three consecutive interstimulus intervals at which participants recognized the stimuli as temporally separated was considered the STDT. The STDT was defined as the average of three STDT values and was entered in the data analysis. The STDT was tested and measured by neurophysiologists who were blinded to the clinical assessment both at the baseline and 8 years later.

### 2.1. Statistical Analysis

We first compared STDT values in dystonic patients upon enrolment with those from a group of age-matched healthy controls using an unpaired sample *t*-test. We then ran a paired sample *t*-test to evaluate changes in clinical scores and STDT values obtained upon enrolment and at the 8-year follow-up assessment in patients with dystonia. To evaluate whether STDT values changed to a different extent in patients with blepharospasm and cervical dystonia across the two assessments, we also ran a between-group repeated measures ANOVA with factor GROUP (blepharospasm versus cervical dystonia) and TIME (two levels: enrolment and 8-year FU). Spearman's correlation coefficient was used to evaluate any relationships between clinical and neurophysiological variables.

## 3. Results

**Figure 1:**
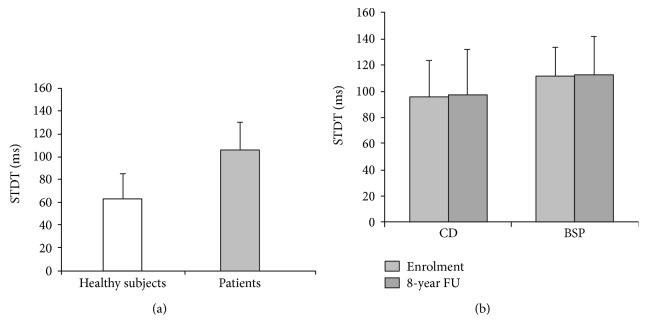
Somatosensory temporal discrimination threshold (STDT) in healthy subjects and in patients with focal dystonias (blepharospasm and cervical dystonia). (a) Mean STDT values (expressed in milliseconds) in healthy subjects and patients with dystonia (whole group). (b) Mean STDT values in patients with blepharospasm and cervical dystonia tested upon enrolment and 8 years later. Bars represent standard deviation.

The unpaired sample *t*-test used to compare STDT values in patients upon enrolment, and healthy subjects showed that STDT values in dystonic patients were higher than those in healthy subjects (*p* < 0.001) (Figure 1).

When we compared the clinical severity scores at the first evaluation with those at the 8-year follow-up evaluation, the paired sample *t*-test revealed a significant increase in disease severity scores in patients (blepharospasm: *p* = 0.007; cervical dystonia: *p* = 0.008) (Table 1). Only 1 patient with cervical dystonia and 7 patients with blepharospasm had spread to other body parts (upper limb in the patient with cervical dystonia and oromandibular dystonia in those with blepharospasm) at the follow-up assessment.

The paired sample *t-*test performed to investigate any changes in STDT values in patients between the baseline evaluation and the 8-year follow-up evaluation showed that STDT values remained unchanged (baseline: 106 ± 25 ms versus 8-year follow-up: 107 ± 32 ms; *p* = 0.83) (Figure 1). Paired sample *t*-test to evaluate whether STDT values from the 8 patients who had clinical signs of spread changed at follow-up showed no significant changes (*p* = 0.85).

Repeated measures ANOVA to evaluate whether STDT changed differently in patients with blepharospasm and cervical dystonia across the two assessments showed neither significant factor TIME (*F* = 0.04, *p* = 0.83) nor significant GROUP × TIME interaction (*F* = 0.001, *p* = 0.97) (Figure 1). Spearman's correlation coefficient did not disclose any significant relationship between STDT values and changes in disease severity scores.

## 4. Discussion

This is the first longitudinal study based on an 8-year follow-up that has evaluated the STDT values during the course of disease in patients with blepharospasm and cervical dystonia. The novel finding of our study is that the abnormally increased STDT values observed in patients with focal dystonias remained unchanged whereas disease severity worsened at the 8-year follow-up assessment.

We took several precautions to ensure that the data we obtained were reliable. The neurophysiologist who tested the patients' STDT was blind to the clinical assessment, and the investigators who performed the clinical assessment were not informed of the purpose of the study. Since botulinum toxin leaves STDT values unchanged in dystonic patients [29], but is known to affect disease severity scores, the assessments both upon enrolment and at follow-up were conducted at least 4 months after the last botulinum toxin injection.

A recent study on the effect of aging on STDT measurements showed that STDT values increases with aging [30]. When they investigated a large sample of healthy subjects, Ramos et al. [30] found that the STDT increases by 0.66 ms every year in subjects older than 65 years. Therefore, the dystonic patients who were aged 60 years upon enrolment and 68 years at the follow-up assessment may have been subject to age-related changes in STDT values. However, our findings are only apparently in contrast to this observation. Indeed, if we bear in mind that the interstimulus interval during STDT testing is increased in 10 ms steps whereas the STDT increases spontaneously by 0.66 ms every year after the age of 60 years, a 10-year age increase would yield a 6.6 ms increase in STDT values. Thus, by increasing the interstimulus interval during STDT testing in 10 ms steps, as we did, age-related increases in STDT values only start having an effect well after 10 years.

Owing to the psychophysical nature of STDT testing, an altered STDT in dystonia may be caused by behavioural/attentional dysfunctions or psychiatric conditions, both of which are known to occur in patients with dystonia [5, 20, 31–33]. Since the STDT relies on the activity of the basal ganglia combined with that of several cortical areas, including the prefrontal areas, and since covert attentional deficits or mood disorders may be responsible for increased STDT values, we expected the dystonia patients' STDT values to change when tested at the follow-up. Our findings showing that the STDT values remained unmodified 8 years after the first assessment contradict this hypothesis.

Previous studies on healthy subjects have demonstrated that STDT values are modulated by plasticity mechanisms in S1 induced by repetitive transcranial magnetic stimulation at the cortical level [13, 22]. A recent study also showed that high-frequency electrical stimulation of an area of skin on a finger improves tactile temporal discrimination and that the improvement is reversed within 24 hours [34]. The authors of that study concluded that the perceptual effects on the STDT they observed are likely to be dependent on plastic changes in the somatosensory cortex, which is in accordance with the concept that the timing of sensory stimuli is, at least in part, encoded in the primary somatosensory cortex. In keeping with this hypothesis, we have recently observed [35] that the temporal discriminative acuity of tactile stimuli is affected by the number of stimuli in the task and suggested that stimulus-driven rapid plasticity is the main mechanism underlying somatosensory temporal encoding in S1.

Investigating the neurophysiological correlates of abnormal somatosensory temporal discrimination in dystonia, Antelmi et al. [25] reported that STDT values were increased in dystonic patients and were associated with reduced suppression of cortical and subcortical paired-pulse somatosensory evoked potentials as well as with a smaller area of the high-frequency oscillation early component. Overall, these findings point to a reduced activity in dystonic patients of the inhibitory interneurons within the primary somatosensory cortex although a possible contribution of altered inhibitory activity in the basal ganglia cannot be excluded.

Our observation that the STDT did not change either in patients with blepharospasm or in those with cervical dystonia, with or without clinical signs of spread, at the follow-up assessment suggests that STDT abnormalities in dystonia are representative of a background alteration in inhibitory mechanisms. We hypothesize that this alteration may be considered a “fingerprint” that remains stable over time and is a predisposing factor of the disease. Since cortical plasticity mechanisms rely on a dynamic balance between excitatory and inhibitory interneurons [36, 37], it is conceivable that altered inhibitory interneuron activity may concur to give rise to other pathophysiological mechanisms in dystonia, such as aberrant cortical plasticity mechanisms [3, 8].

Our findings showing that STDT values are unrelated to the severity of motor disturbances and that they do not change after 8 years despite the progression in dystonia severity suggest that abnormal STDT is not a marker of disease progression but is an endophenotypic marker of the disease. On the same line, STDT changes are present when dystonic features are not yet manifested in patients with increased blinking, a condition now considered to be a prodromal manifestation of blepharospasm [38, 39]. Different from dystonia in other basal ganglia conditions, like Parkinson's disease, STDT changes reflect dopaminergic depletion [40, 41] and disease progression [42, 43].

A limitation of the present study may be the lack of a control group at follow-up. However, since STDT values in dystonic patients were already altered at baseline and since STDT values in dystonic patients remained unmodified at follow-up, we believe that the lack of a control group at follow-up unlikely affects the interpretation of our findings.

In conclusion, the results of our study showing that STDT abnormalities in dystonia remain unmodified during the course of the disease suggest that the abnormal activity of inhibitory interneurons does not deteriorate further as the disease progresses.

## Figures and Tables

**Table 1:** 

Pts	Gender	Age at the enrolment (years)	Duration at the enrolment (years)	Type of dystonia	Disease severity^∗^ at baseline	Disease severity 8-year FU
1	F	73	7	BPS	3	3
2	F	58	8	BPS	2	3
3	F	74	17	BPS	3	3
4	M	64	2	BPS	3	3
5	F	76	30	BPS	1	2
6	F	66	10	BPS	3	3
7	F	73	20	BPS	1	3
8	M	73	11	BPS	2	3
9	M	58	6	BPS	3	3
10	M	72	9	BPS	3	3
11	M	73	22	BPS	2	2
12	M	50	3	BPS	2	3
13	F	73	12	BPS	2	2
14	F	82	25	BPS	1	3
15	F	51	23	BPS	2	3
16	F	51	2	CD	11	23
17	F	49	12	CD	18	16
18	F	66	12	CD	8	17
19	F	73	2	CD	12	15
20	F	44	21	CD	16	20
21	F	51	2	CD	10	29
22	F	53	7	CD	9	19
23	M	35	12	CD	12	16
24	M	42	12	CD	15	17
25	F	50	15	CD	12	16

BSP: blepharospasm; CD: cervical dystonia; ^∗^disease severity in BSP patients represents scores on a three-point scale whereas in patients with CD it represents scores at the Toronto Western Spasmodic Torticollis Rating Scale (TWSTRS); FU: follow-up.
